# A novel luciferase fusion protein for highly sensitive optical imaging: from single-cell analysis to in vivo whole-body bioluminescence imaging

**DOI:** 10.1007/s00216-014-7917-2

**Published:** 2014-06-24

**Authors:** Laura Mezzanotte, Vicky Blankevoort, Clemens W. G. M. Löwik, Eric L. Kaijzel

**Affiliations:** Department of Radiology, Experimental Molecular Imaging, Leiden University Medical Center, Albinusdreef 2, 2333 ZA Leiden, The Netherlands

**Keywords:** Fluorescence/luminescence, Cell systems/single-cell analysis, Bioanalytical methods

## Abstract

Fluorescence and bioluminescence imaging have different advantages and disadvantages depending on the application. Bioluminescence imaging is now the most sensitive optical technique for tracking cells, promoter activity studies, or for longitudinal in vivo preclinical studies. Far-red and near-infrared fluorescence imaging have the advantage of being suitable for both ex vivo and in vivo analysis and have translational potential, thanks to the availability of very sensitive imaging instrumentation. Here, we report the development and validation of a new luciferase fusion reporter generated by the fusion of the firefly luciferase Luc2 to the far-red fluorescent protein TurboFP635 by a 14-amino acid linker peptide. Expression of the fusion protein, named TurboLuc, was analyzed in human embryonic kidney cells, (HEK)-293 cells, via Western blot analysis, fluorescence microscopy, and in vivo optical imaging. The created fusion protein maintained the characteristics of the original bioluminescent and fluorescent protein and showed no toxicity when expressed in living cells. To assess the sensitivity of the reporter for in vivo imaging, transfected cells were subcutaneously injected in animals. Detection limits of cells were 5 × 10^3^ and 5 × 10^4^ cells for bioluminescent and fluorescent imaging, respectively. In addition, hydrodynamics-based in vivo gene delivery using a minicircle vector expressing TurboLuc allowed for the analysis of luminescent signals over time in deep tissue. Bioluminescence could be monitored for over 30 days in the liver of animals. In conclusion, TurboLuc combines the advantages of both bioluminescence and fluorescence and allows for highly sensitive optical imaging ranging from single-cell analysis to in vivo whole-body bioluminescence imaging.

**Fig:**
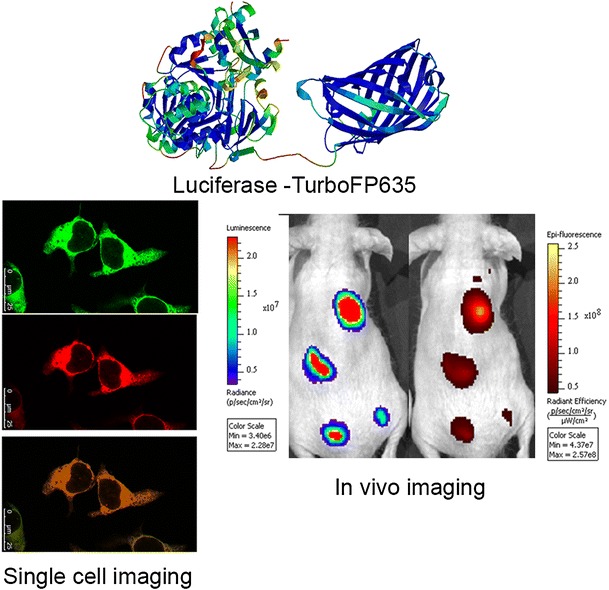
Optical imaging using TurboLuc fusion reporter protein

Optical imaging using TurboLuc fusion reporter protein

## Introduction

From its early development in the 1990s, in vivo optical imaging (OI) demonstrated a growing interest by the scientific community and a large number of applications in many research areas from plant research to biochemical, biopharmaceutical, and biomedical sciences [1]. This is due to the fact that OI techniques are easy to perform, with low cost and high throughput. Optical imaging is usually performed by the use of reporter genes, appropriate substrates for luminescent reactions or luminescent compounds (e.g., fluorescent probes) in order to understand the dynamics of in vivo molecular events in cells or in small animals [2]. In the last decades, optical reporter genes for both fluorescence (FLI) or bioluminescence imaging (BLI) have been extensively employed to image molecular pathways at the transcriptional level or integrated into the genome for long-term expression and for the development of transgenic animals [3–5]. In addition, optical reporter genes have been modified to ameliorate luminescent properties of the proteins like the photo- and thermostability, maturation time, emission spectra, and quantum yields. This resulted in improved in vivo sensitivity and stability characteristics of optical reporters that enabled multiple readouts and (development of) high-throughput assays. In research questions that warrant monitoring molecular events from single cells to small animals, strategies that combine the application of both fluorescence and bioluminescence are preferable. At a cellular level, fluorescent proteins provide a high spatial and temporal resolution and good contrast for delineating cell structures, making FLI explicitly suitable for (single) cell imaging, while BLI allows for the noninvasive study of ongoing biological processes in small laboratory animals. Reporter genes can be co-expressed in cells or animal by two strategies: the first one is molecular cloning of the reporter genes into bicistronic or tricistronic constructs that allow the simultaneous transcription of different genes while the second is development of fusion proteins. The second strategy is preferable when there is a need to co-localize the reporters in the cell during experiments. Moreover, the chimerae may be more stable or more soluble in the host cell than the native proteins would be. Dual or triple fusion reporter proteins have been developed to image cancer or stem cells and employed Renilla luciferase or firefly luciferase (*Photinus pyralis*) fused to green fluorescent proteins (GFPs) or red fluorescent proteins (RFPs) [6–8]. In the last few years, new far-red fluorescent proteins that are bright and suitable for cell and deep tissue imaging have been developed. Among these, TurboFP635 (scientific name Katushka) shows emission maxima at 635 nm and is seven- to tenfold brighter compared to the spectrally close HcRed or mPlum fluorescent proteins. The protein is also characterized by fast maturation as well as high pH stability and photostability [9]. The high photostability offers advantages for stimulated emission depletion (STED) microscopy by which super-resolution is achieved in studies to elucidate protein structure analysis at sub-organelle level. These unique characteristics make TurboFP635 an attractive component for biosensors in cells, for deep tissue imaging, and long-term intravital imaging [10]. Also, for whole-body optical imaging in small laboratory animals, TurboFP635 has shown to be superior to other reported FPs [11].

Recently, our group and others confirmed the superior bioluminescent imaging performance of the Luc2 reporter gene for in vivo imaging using a single reporter as a result of its high expression level in mammalian cells, stability, spectrum of emission at 37 °C, and quantum yield of the enzymatic reaction [12, 13]. Therefore, the combination of TurboFP635 characteristics with the luciferase ones will offer advantages in many bioanalytical applications like STED microscopy, long-term fluorescence intravital imaging of subcellular structures in live mice [14], intravital fiber-optic fluorescence imaging [15], and whole-body optical imaging [11].

The resulted protein showed no altered bioluminescence of fluorescence properties compared to the individual counterparts, and it is expressed in mammalian cells as a fused reporter. Expression of the TurboLuc fusion protein was characterized by fluorescence microscopy and in vivo optical imaging. Subsequently, the fusion reporter gene was cloned into a minicircle vector and used for hydrodynamic tail-vein injection in mice to analyze the sustained expression of the fusion protein. Minicircles are episomal DNA vectors that are produced as circular expression cassettes devoid of any bacterial plasmid DNA backbone and have been shown to greatly increase the efficiency of transgene expression in various in vitro and in vivo studies [16, 17]. Their application is growing: minicircles have recently been applied in human iPS cell technology and are of great promise as a nonintegrating method in stem cell engineering in a clinical setting [18, 19]. This novel luciferase fusion protein was revealed to be a unique and useful tool for combined single-cell analysis and sensitive in vivo imaging in preclinical research.

## Materials and methods

### Vector construction

The pGL3Luc2 vector was created by excision of luc2 gene with NcoI and XbaI from pGL4.10 and subcloned into a pGL3-basic vector (Promega, Leiden, The Netherlands). The mammalian expression vector pTurboFP635-N encoding far-red fluorescent protein TurboFP635 (Evrogen, Moscow, Russia) was used for the fusion of Luc2 reporter gene at the N-terminal region of the TurboFP635. Briefly, Luc2 gene was amplified from pGL4.10 vector using the sense primer 5′-CCGCTAGCAATGGAAGATGCCAAAAACAT-3′ with a NheI restriction site and antisense primer 3′-CGTGACTGCTTGCCGCCCTTCTTGGCCTT-5′ with a SalI restriction site. Subsequently, the fragment was cloned in the multiple cloning site of the pTurboFP635-N, allowing the generation of the fusion protein. The new vector was named TurboLuc. Then, the fusion protein TurboLuc was amplified using the sense primer 5′CTCTAGAGCAATGGAAGATGCCAAAAACAT-3′ with an XbaI restriction site and antisense primer 3′-TGAATTCCATCAGCTGTGCCCCAGTTTGCTA-5′ with an EcoRI site. The fragment was subcloned into pMN502A-1, a parental minicircle vector purchased from SBI (System Bioscience, Mountain View, CA, USA), and the resulting vector was called pMNTurboLuc. The minicircle vector, called MINI-pMNTurboLuc, was then purified and isolated following the protocol of the MC-easy minicircle DNA production kit (System Bioscience).

### Cell culture and transfection

Human embryonic kidney cells (HEK-293) were cultured in DMEM GlutaMAX (Invitrogen, Darmstadt, Germany) supplemented with 10 % FCS (Sigma, St. Louis, USA), 1 % penicillin–streptomycin (Sigma), and 2 mM l-glutamine (PAA). The cells were grown at 37 °C in a 5 % CO_2_-humidified incubator. Cells (2 × 10^4^/well) were seeded in a 96-well plate and transfected with 0.1 μg of pTurboLuc or pTurboFP635 for analysis of fluorescence signals and immunofluorescence staining. For testing the expression of TurboLuc in cells using a minicircle vector, cells were seeded in a 24-well plate (5 × 10^4^ cells/well) and transfected with 1 μg of parental pMNTurboLuc or MINI-pMNTurboLuc by using the reagents and following the procedure described in the MC-easy minicircle DNA production kit’s protocol.

### Western blot analysis of luciferase protein

To confirm the presence of the fusion protein, we performed Western blot analysis of cells transfected with pGL3Luc2, pTurboLuc, and pTurboFP635-N. Two independent experiments were carried out. Briefly, HEK-293 cells (5 × 10^5^/well) were seeded in six-well plates for 24 h, then transfected with 3.3 μg of plasmid DNA using FugeneHD transfection reagent (Promega). After 24 h of treatment, cells were collected, and the total amount of protein of each sample was determined by a Pierce BCA protein assay kit (Thermo Scientific, Rockford, USA). Fifteen microgram of whole cell extract was applied to a 10 % SDS-PAGE and transferred onto a nitrocellulose membrane. After washing, the membrane was blocked in Tris–phosphate-buffered saline (TPBS) 5 % bovine serum albumin (BSA) and incubated with an anti-Luc rabbit polyclonal antibody (Luciferase antibody 20R-1419 from Fitzgerald Industries, Acton, MA, USA) diluted with 1:500 in TPBS, overnight at room temperature. The blots were washed, exposed to HRP-conjugated secondary antibodies for 1 h, and finally detected using an enhanced chemiluminescence (ECL) reagent (Thermo Scientific). Detection of ECL signals was performed with ChemiDoc™ MP System (Biorad, Hercules, CA, USA).

### Immunofluorescence

Transfected HEK-293 cells were fixed in precooled 4 % paraformaldehyde at room temperature for 10 min and washed three times with PBS. The main procedures for immunofluorescence staining were as follows: (1) incubation with 0.1 % Triton X-100 at room temperature for 30 min; (2) incubation with 5 % BSA at room temperature for 30 min; (3) incubation with the primary antibody at 4 °C for 24 h (the primary antibody used was rabbit anti-luciferase antibody (20R-1419) at a dilution of 1:500); (4) the secondary antibody used was goat anti-rabbit IgG fluorescein isothiocyanate (FITC), at 1:100, with incubation at room temperature in the dark for 2 h; (5) dilution of DAPI at 1:1,000 in PBS at room temperature for 5 min; and (6) mounting of slides with Fluoromount (Sigma; F4680), analyzed with fluorescence microscopy.

### Optical imaging

#### In vitro

Luciferase emission spectrum was determined as follows: the medium in wells containing transfected cells was replaced with phosphate-buffered saline, and d-luciferin (Synchem, Germany) was added at a final concentration of 0.5 mM. Luciferase activity was measured using an IVIS Spectrum (Caliper, Alameda, CA, USA). The instrument stage was kept at 37 °C, and the imaging setup was field of view (FOV) C. Light output was measured using an open filter and a series of band pass filter (20 nm) ranging from 500 to 700 nm each for 5 s, 5 min after substrate addition to live cells. For in vitro fluorescence measurements, the instrument was set with an excitation filter of 570 nm and an emission filter of 640 nm. All the data are expressed in photon flux and analyzed with Living Image Software 4.0 (Caliper, Alameda, CA, USA). For the analysis of bioluminescence from cells transfected with pMNTurboLuc or MINI-pMNTurboLuc, cells were analyzed at different days after transfection using One-Glo luciferase assay substrate (Promega) and a plate luminometer (SpectraMax L luminometer, Molecular Devices, Sunnyvale, CA, USA).

#### In vivo

All animal experiments were approved by the local committee for animal health, ethics, and research of the Leiden University Medical Center. In the first set of experiments, different amounts of transfected cells (5 × 10^2^, 5 × 10^3^, 5 × 10^4^, 1 × 10^5^, 2.5 × 10^5^, and 5 × 10^5^) were implanted subcutaneously in a 50-μl volume. BALB/c nu/nu mice (*n* = 6) were used, and data are presented as mean and standard deviation of luminescent signals.

In the second set of experiments, a hydrodynamic injection of 10 μg of MINI-pMNTurboLuc (dissolved in 2 ml of PBS and injected in tail vein in 5–8 s) was performed in mice (*n* = 3) to evaluate the expression in deep organs such as the liver. Light emission was collected using the IVIS Spectrum (Caliper, Alameda, CA, USA). For fluorescence measurement, an excitation filter at 570 nm and an emission filter at 640 nm were used. d-Luciferin (150 mg/kg) was injected intraperitoneally, and image acquisition started 10 min later using a FOV C and a 30-s acquisition time.

## Results and discussion

### Construction of vectors and cell imaging

**Fig. 1:**
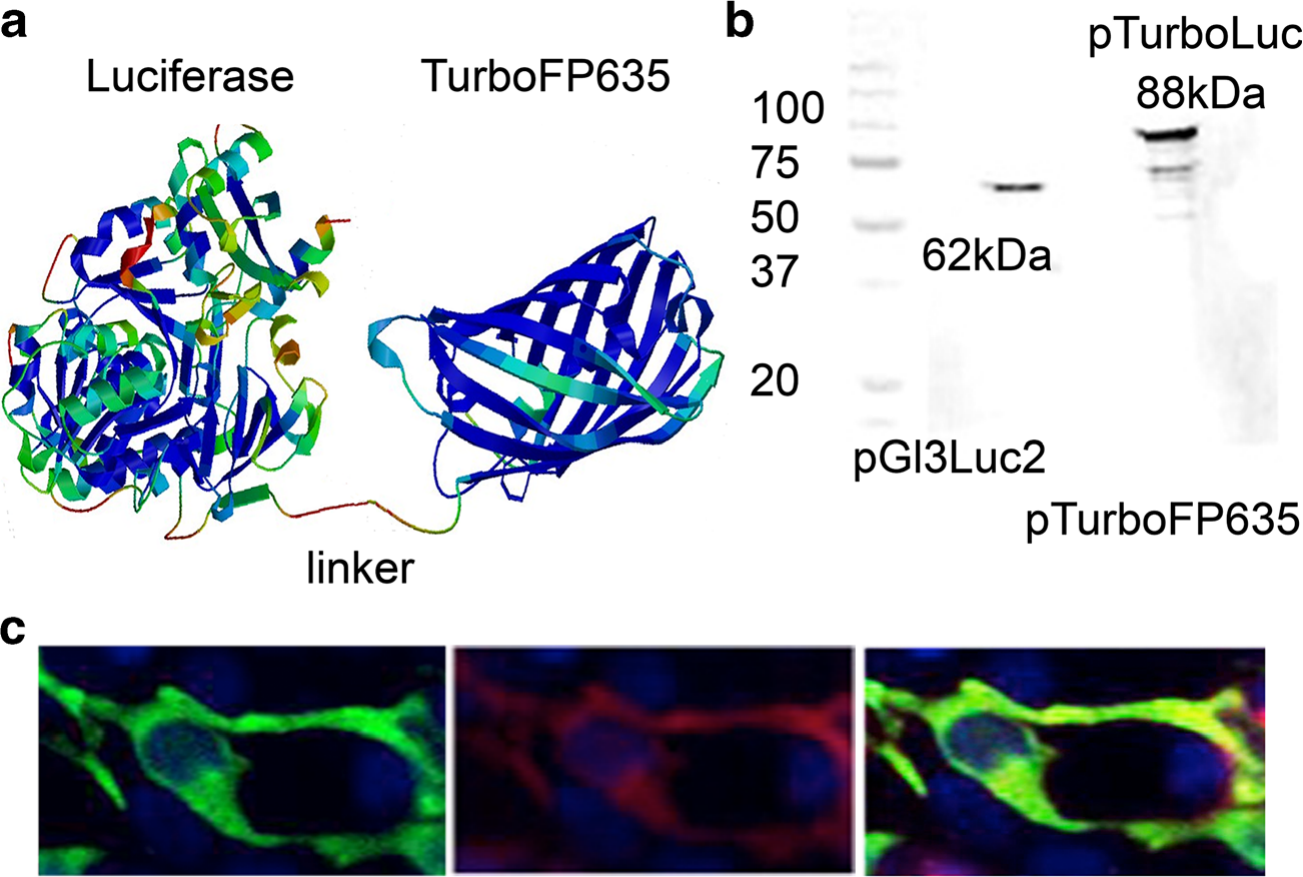
**a** Predicted protein configuration of TurboLuc based on the sequence of amino acid residues [24]. **b** Western blot analysis of whole extracts of cells transfected with pGL3Luc2, pTurboFP635, and pTurboLuc using an anti-Luc antibody showing the presence of the fusion protein of 88 kDa. Extracts from pGL3Luc2-transfected cells served as a positive control showing the expression of the regular 62-kDa protein. Extracts from pTurboFP635-expressing cells were used as a negative control. **c** Immunofluorescence analysis of cells using an anti-luciferase primary antibody and a FITC-labeled secondary antibody. Cells have been imaged with a blue filter for DAPI (nuclei), a green filter for FITC (Luc2), and a red filter for TurboFP635. Colocalization of red and green signals demonstrates the expression of the fusion protein

**Fig. 2:**
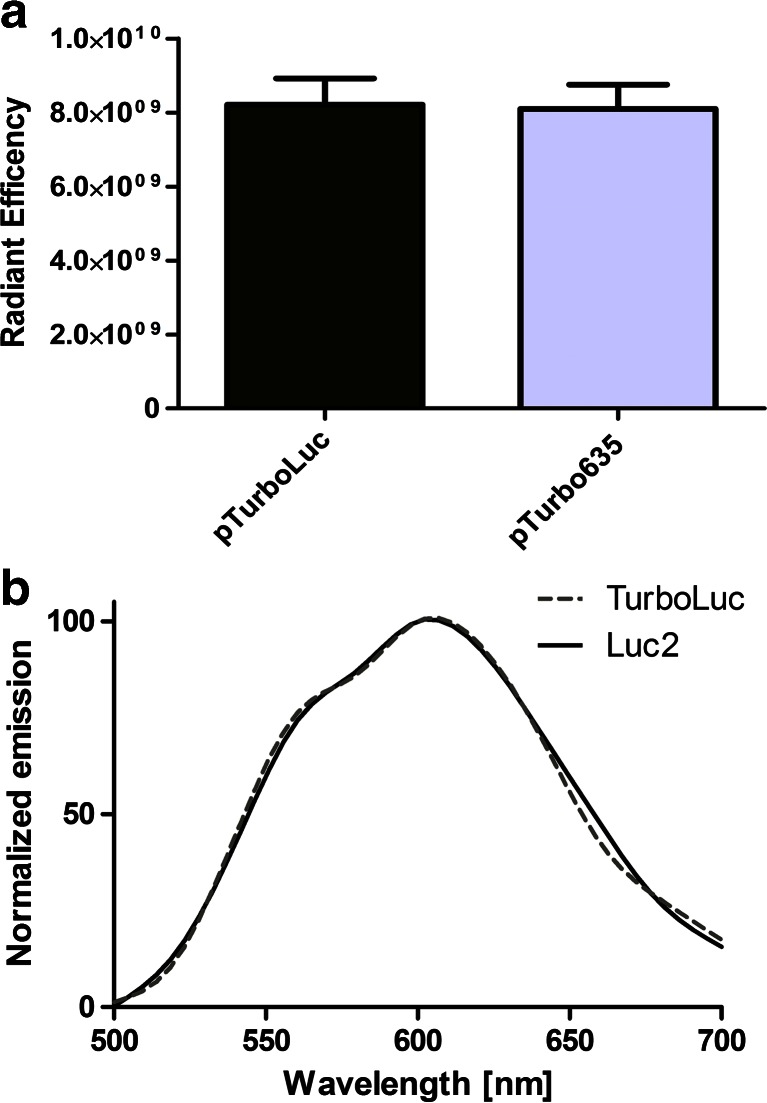
**a** Fluorescent signal output of cells expressing either TurboLuc or TurboFP635 showed no significant differences. **b** Comparable normalized bioluminescence emission spectra of HEK293 cells expressing either TurboLuc or Luc2 at 37 °C

The choice of the amino acid linker is of great importance in the construction of stable and bioactive fusion proteins. In previous reports, generation of luciferase fusion proteins has been performed by the addition of long linkers at the C-terminal end of the luciferase gene [8]. In our construct, a linker of 14 amino acids (QSTVPRARDPPVAT) connecting the C-terminal region of the Luc2 gene to the N-terminal region of the TurboFP635 protein (Fig. 1a) appeared to be enough to enable stability of the protein in vivo. Western blot analysis was carried out to confirm the correct expression of the fusion protein by using an anti-luciferase antibody (Fig. 1b). Whole protein extracts derived from cells transfected with pTurboLuc revealed the presence of an 88-kDa protein band as a result of the fusion of Luc2 protein (62 kDa) and TurboFP635 (26 kDa). Immunofluorescence staining on transfected cells using an anti-luciferase antibody confirmed the correct expression of the TurboLuc protein in cells (Fig. 1c) as the bioluminescent and fluorescent signals co-localize. Next, the fluorescence and bioluminescence emission characteristics of the fusion protein were evaluated. HEK-293 cells were transfected with an equal amount of pTurboLuc vector or the parental pTurboFP635, and fluorescence emission was analyzed 24 h later. As shown in Fig. 2a, both vectors revealed a similar fluorescent signal output in transfected live cells showing that the fusion of the reporter did not affect fluorescent emission. Moreover, the measured luciferase spectra of pTurboLuc and Luc2 were similar in cells at 37 °C (Fig. 2b). These data support the assumption that the created TurboLuc fusion protein will most probably retain the same efficiency as the native luciferase and fluorescent TurboFP635 counterparts when used in (single) cell-based assays or in vivo imaging. This is of particular importance since in some cases the fusion of a fluorescent protein at the C-terminus of the firefly luciferase has been shown to interfere with a correct fold and function of the fusion protein when highly expressed in certain cell types (e.g., neurons) or during aging and disease [20–22].

### Evaluation of in vivo fluorescence and bioluminescence sensitivity imaging

**Fig. 3:**
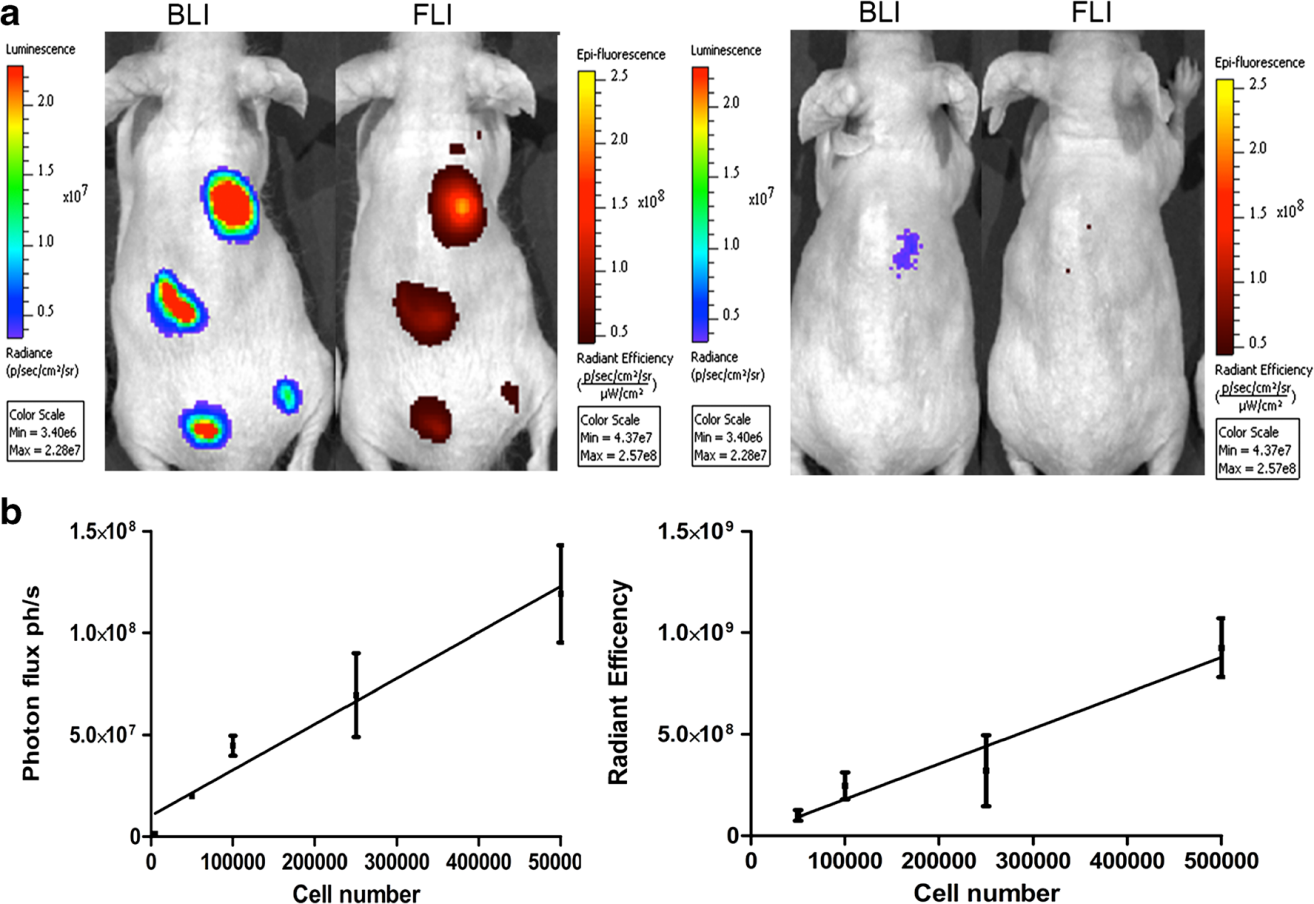
**a** Representative bioluminescence and fluorescence images of a mouse subcutaneously injected with 5 × 10^4^, 1 × 10^5^, 2.5 × 10^5^, and 5 × 10^5^ HEK-293 cells expressing TurboLuc (*on the left*) and 5 × 10^2^, 5 × 10^3^ cells (*on the right*). Bioluminescence measurement allowed detecting 5 × 10^3^ cells while fluorescence imaging, 5 × 10^4^ cells. **b** Graph showing the linear correlation between luminescent signals and the number of injected cells

To test the sensitivity of TurboLuc in vivo, pTurboLuc-expressing HEK-293 cells were subcutaneously injected in nude mice in an amount ranging from 5 × 10^2^–5 × 10^5^ (Fig. 3a). Bioluminescence signals were proportional to the amount of injected cells (linear correlation coefficient *R* ^2^ = 0.98), and the detection limit was around 5 × 10^3^ cells (Fig. 3b). Fluorescence measurements also showed a linear correlation between signal emission and number of injected cells (*R* ^2^ = 0.86). However, the limit of detection was 1 order of magnitude higher (5 × 10^4^ cells). These results imply that for superficial imaging of more than 5 × 10^4^ cells, the fluorescence imaging can give an indication of the presence of the cells with no need to use a luminescent substrate. However, most applications nowadays require the visualization of cells or molecular events in deeper tissue.

### Evaluation of efficiency of TurboLuc for deep tissue imaging

To evaluate TurboLuc imaging in deep tissue, the fusion reporter gene was cloned into a parental minicircle vector (MINI-pMNTurboLuc) to perform hydrodynamics-based transfection of animals. Minicircle vectors allow for sustained expression of a reporter as a result of the elimination of the bacterial sequences from the parental plasmid that interfere with expression. Hydrodynamic injection of DNA allows for the expression of naked DNA mostly in the liver, a deep tissue.

**Fig. 4:**
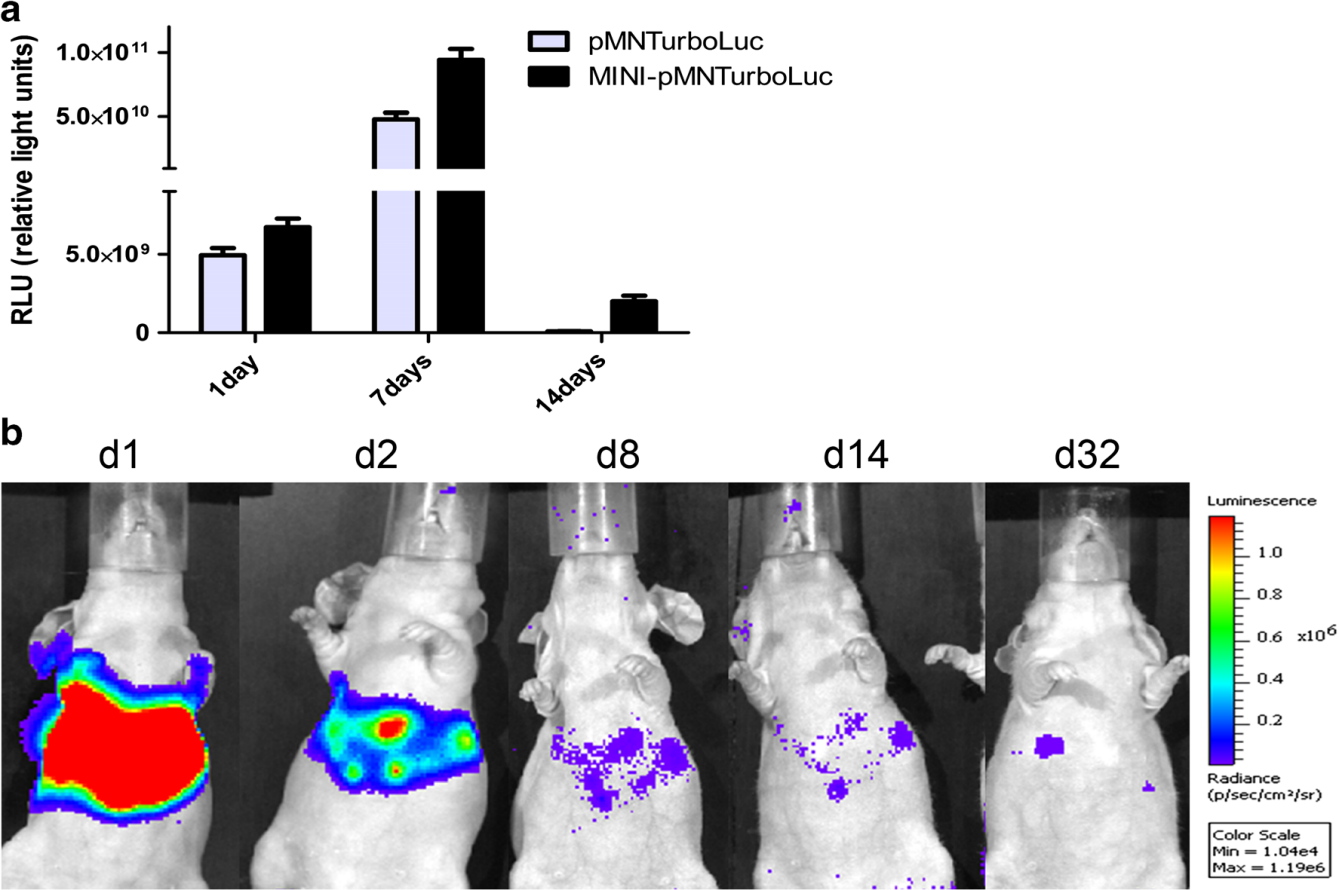
**a** Light emission from 2 × 10^4^ lysed cells on different days after transfection with pMNTurboLuc or the minicircle vector encoding TurboLuc. Light emission of pMNTurboLuc is higher at day 7, while expression of the minicircle vector encoding TurboLuc (MINI-pMNTurboLuc) lasts for more than 14 days. **b** Representative images of sustained Luc expression in the liver of an animal after hydrodynamic injection of MINI-pMNTurboLuc

First, pMNTurboLuc vector was compared with MINI-pMNTurboLuc for bioluminescence imaging during 14 days in vitro. As shown in Fig. 4a, the comparison between light outputs of the transfected cells showed the retained expression after 7 and 14 days, while the signal obtained with the pMNTurboLuc was lower at 7 days and was not detectable after 14 days. In vivo bioluminescence expression of the minicircle vector MINI-pMNTurboLuc lasted for over 32 days in the liver as shown in Fig. 4b. However, the fluorescent signals could not be detected.

TurboFP635 is one of the brightest red fluorescent proteins [11]. The TurboLuc fusion protein can therefore be considered as an optimized/superior reporter fusion protein in terms of brightness and quantum yield and stability with a large spectrum of applications in preclinical research both in vitro and in vivo. Smart new luciferase–fluorescent protein fusions can also be applied in other imaging purposes. Recently, Saito K et al. developed a luciferase fusion reporter, which is a chimera of enhanced Renilla luciferase and Venus [23]. Apart from the main application of single-cell imaging, the authors also showed some application for whole-body imaging. Since Renilla luciferase requires coelenterazine as a substrate, it will be possible to combine it with the developed TurboLuc reporter enzyme that requires d-luciferin. This will enable dual-color bioluminescence using the two different substrates. Moreover, since Venus mostly emits green light while Turbo635 has a far-red emission, dual-color fluorescence can also be performed, enabling a number of ex vivo applications (e.g., immunofluorescence and microscopy on single cells). Recently, a fusion of luciferase with a near-infrared protein combined near-infrared imaging with optoacoustic imaging. This enabled multimodality imaging and led to a better in vivo resolution [24, 25]. Moreover, TurboLuc can be subsequently fused to a thymidine kinase (TK) reporter gene to allow positron emission tomography (PET) imaging as an improved version of the existing triple fusion reporter published by Ray et al. [26] in 2007.

There are also opportunities to use this fusion reporter gene for the generation of transgenic mice. Recently, Ray and colleagues developed transgenic mice expressing a triple fusion reporter in which Luc2 was fused to dTomato and TK gene [27]. These mice ubiquitously expressed the triple fusion reporter and could be used for multimodality imaging purposes. A Cre-reporter transgenic animal expressing TurboFP635 has been reported, and transgenic animals expressing the luciferase fused to GFP are also available [28–30]. The expression of TurboFP635 or luciferase in these mice showed no toxicity and low immunogenicity. This opens up the possibility to develop transgenic mice employing TurboLuc for highly sensitive imaging: these mice could be employed as a source of luminescent cells (e.g., isolation of organs or mouse stem cells) or as mouse strains for crossing with other mouse models of disease. However, possible negative effects of TurboLuc expression on embryo development, for the generation of transgenics, are largely unknown and need to be determined.

## Conclusion

In this study, we report the generation and validation of a new fusion protein that is a chimera between firefly luciferase (Luc2) and TurboFP635 named TurboLuc. The expressed fusion protein retained the good sensitivity of the Luc2 reporter, and it showed no altered fluorescence emission. It can be combined with other existing reporters for multicolor bioluminescence and fluorescence imaging. Therefore, the new reporter protein shows great potential for imaging molecular events from single cells to living intact animals.
